# The Role of Renin–Angiotensin System in Diabetic Cardiomyopathy: A Narrative Review

**DOI:** 10.3390/life13071598

**Published:** 2023-07-21

**Authors:** João Pedro Thimotheo Batista, André Oliveira Vilela de Faria, Thomas Felipe Silva Ribeiro, Ana Cristina Simões e Silva

**Affiliations:** 1 Laboratório Interdisciplinar de Investigação Médica, Faculdade de Medicina, Universidade Federal de Minas Gerais (UFMG), Belo Horizonte 30130-100, MG, Brazil; timotio.batista@gmail.com (J.P.T.B.); andrevfaria@gmail.com (A.O.V.d.F.); thomas.felipe.silva@gmail.com (T.F.S.R.); 2 Departamento de Pediatria, Faculdade de Medicina, Universidade Federal de Minas Gerais (UFMG), Belo Horizonte 30130-100, MG, Brazil

**Keywords:** renin–angiotensin system, diabetic cardiomyopathy, angiotensin II, angiotensin-(1-7), oxidative stress

## Abstract

Diabetic cardiomyopathy refers to myocardial dysfunction in type 2 diabetes, but without the traditional cardiovascular risk factors or overt clinical atherosclerosis and valvular disease. The activation of the renin–angiotensin system (RAS), oxidative stress, lipotoxicity, maladaptive immune responses, imbalanced mitochondrial dynamics, impaired myocyte autophagy, increased myocyte apoptosis, and fibrosis contribute to diabetic cardiomyopathy. This review summarizes the studies that address the link between cardiomyopathy and the RAS in humans and presents proposed pathophysiological mechanisms underlying this association. The RAS plays an important role in the development and progression of diabetic cardiomyopathy. The over-activation of the classical RAS axis in diabetes leads to the increased production of angiotensin (Ang) II, angiotensin type 1 receptor activation, and aldosterone release, contributing to increased oxidative stress, fibrosis, and cardiac remodeling. In contrast, Ang-(1-7) suppresses oxidative stress, inhibits tissue fibrosis, and prevents extensive cardiac remodeling. Angiotensin-converting-enzyme (ACE) inhibitors and angiotensin receptor blockers improve heart functioning and reduce the occurrence of diabetic cardiomyopathy. Experimental studies also show beneficial effects for Ang-(1-7) and angiotensin-converting enzyme 2 infusion in improving heart functioning and tissue injury. Further research is necessary to fully understand the pathophysiology of diabetic cardiomyopathy and to translate experimental findings into clinical practice.

## 1. Introduction

Heart failure (HF) is a growing health issue that consists in structural and consequent functional changes in cardiac tissue related to a myriad of risk factors. Approximately 64 million people in the world have HF, and its prevalence is increasing [1]. A recent epidemiological study including 40 different countries evaluated the differences in HF etiology, treatment, and outcomes according to levels of economic development [2]. The study evaluated data from 23,341 individuals with a mean of 63.1 years and a predominance of the male gender (60.9%). Diabetes contributed to HF in 7209 individuals (30.9%) [2]. 

Increasing rates of aging, obesity, and diabetes are significantly responsible for the upsurge in HF and the associated morbidity and mortality. Importantly, patients with diabetes experience significantly worse clinical outcomes than patients without diabetes, especially related to HF and cardiac remodeling [3]. In these individuals, the term “diabetic cardiomyopathy” refers to myocardial dysfunction in the absence of traditional cardiovascular risk factors (hypertension, dyslipidemia) or overt clinical atherosclerosis and valvular disease. The current literature shows that diabetes has a clear and direct impact on interstitial fibrosis, resulting in reduced left-ventricular compliance and diastolic dysfunction [4]. The clinical course of cardiac dysfunction in diabetes ranges from asymptomatic heart diseases, such as left-ventricular fibrosis and diastolic dysfunction, to severe diastolic HF with a normal ejection fraction and reduced ejection fraction [5,6].

The current literature presents consistent data on the association between hyperglycemia, insulin resistance, and glucose intolerance and the onset and development of diabetic cardiomyopathy. However, the exact molecular mechanisms and the regulatory factors involved in this relationship are still largely unknown. The risk factors related to type 2 diabetes (T2DM) and the installment and progression of complications are multiple and organized in a complex, multidirectional way. The activation of the renin–angiotensin system (RAS), oxidative stress, lipotoxicity, maladaptive immune responses, imbalanced mitochondrial dynamics, impaired myocyte autophagy, increased myocyte apoptosis, and fibrosis are all factors in the pathogenesis of diabetic cardiomyopathy, which ultimately results in left-ventricular diastolic and systolic dysfunction [7,8,9,10,11,12]. Of crucial importance is RAS activation by hyperglycemia in circulation and cardiac tissue. 

There is growing evidence supporting the harmful effects of the RAS, especially associated with angiotensin II (Ang II) actions on cardiac remodeling and insulin resistance. The heptapeptide angiotensin-(1-7) (Ang-(1-7)) opposes the actions of Ang II and has a protective profile in the cardiovascular system [9,13,14,15,16]. Indeed, RAS actions are the final results of a balance between two axes [17]. The angiotensin-converting enzyme (ACE) Ang II and the angiotensin type 1 (AT1) receptor form the classical RAS axis, whereas the enzyme ACE2 [18,19], Ang-(1-7), and its G-coupled protein Mas receptor [20] are the components of the alternative or counterregulatory RAS axis. Some clinical trials have investigated the role of the inhibition of the classical RAS axis in diabetic patients. In this regard, the Micro-HOPE (Microalbuminuria, Cardiovascular and Renal Outcomes–Heart Outcomes Prevention Evaluation) study, which included 3577 patients with diabetes and chronic kidney disease, found that the ACE inhibitor ramipril reduced the risk of major adverse cardiovascular events by 25% [21].

This review summarizes the studies that address the link between cardiomyopathy and the RAS in humans and presents proposed pathophysiological mechanisms underlying this association.

## 2. Methods

This is a narrative review summarizing scientific findings on the relationship between the RAS and diabetic cardiomyopathy. The authors searched articles in PubMed, Web of Science, and Scopus, using many combinations of the following search terms: “renin angiotensin system” or “renin angiotensin aldosterone system” or “angiotensin II” or “angiotensin-(1-7)” and “diabetes” or “type 2 diabetes” and “heart diseases” or “cardiovascular diseases or “cardiomyopathy”. The articles were selected based on the relevance of the findings for the main topics of this narrative review.

## 3. The Renin–Angiotensin System and the Heart

### 3.1. Local Cardiac RAS

The heart possesses its local renin–angiotensin system, just like other vital organs. It exerts autocrine and paracrine actions to regulate physiological processes. The unbalance of the local RAS can result in different diseases [22]. Some studies advocate against the existence of a local RAS in the heart tissue, mainly supported by evidence suggesting that renin is uptaken from the peripheral circulation [23] and that the renin levels in the heart fall to undetectable values after bilateral nephrectomy [24]. However, there are enough data to support that the heart is capable of synthesizing angiotensin precursors [22]. Two experimental studies [25,26] targeting angiotensinogen messenger RNA (mRNA) synthesis in human heart tissue proved that angiotensinogen is synthesized in the human heart. In fact, an RAS blockade resulted in upregulated plasma Ang-(1-7) and ACE2 mRNA in heart tissue [26]. In the context of the RAS and diabetic cardiomyopathy, specific microRNAs (miRNAs) have been found to be dysregulated and associated with disease progression. For example, miRNA-21, miRNA-29, miRNA-133, and miRNA-155 have been linked to the regulation of RAS components and pathways involved in cardiac remodeling, fibrosis, inflammation, and hypertrophy. These miRNAs can target and modulate the expressions of genes related to the RAS pathway, including ACE, AT1 receptor, and other key signaling molecules [27,28]. Considering these findings, the cardiac RAS is probably the result of both local production and systemic circulation.

The local cardiac RAS is distinct from the circulating RAS. The components of the RAS can be independently produced in heart tissue. The local cardiac RAS plays a critical role in cardiac function, remodeling, and adaptation to physiological and pathological conditions [29]. Several studies have investigated the presence of renin mRNA in the heart using various techniques. Some studies detected low levels of renin mRNA [30,31], whereas others were unable to replicate these findings [32]. Transgenic mice carrying a genomic human renin construct did not show cardiac renin expression [33], while transgenic rats carrying a genomic construct of the mouse Ren-2 gene expressed high levels of renin mRNA in the heart [34]. These findings suggest that the heart may be a site of extrarenal renin production in certain species. While the existence and significance of renin expression in the heart have been subject to debate, the presence of renin mRNA in the heart and its functional implications continue to be investigated. The local cardiac RAS, including renin production and receptor-mediated actions, may contribute to the generation of Ang II and play a role in cardiac physiology and pathophysiology [35].

The existence of local cardiac angiotensin signaling independent of renin has been a subject of debate and investigation. While the classical RAS involves the enzymatic action of renin on angiotensinogen to produce Ang I, followed by the conversion of Ang I into Ang II by an ACE, some studies have suggested the possibility of local cardiac angiotensin signaling pathways that do not require renin [36,37]. Several lines of evidence support the concept of local cardiac angiotensin signaling independent of renin. Ang II can be produced within the heart independently of systemic renin. Other enzymes, including chymase and cathepsin G, have been identified in the heart and shown to have the ability to convert Ang I into Ang II without the involvement of renin [36]. In addition to the production of Ang II, local cardiac enzymes may also metabolize Ang I into various bioactive peptides. For example, ACE2 can convert Ang I into angiotensin-(1-9), which can then be further metabolized into angiotensin-(1-7) [38]. These peptides have distinct physiological effects and may contribute to local angiotensin signaling in the heart. However, the functional significance of these findings to cardiac angiotensin signaling is still a topic of investigation.

### 3.2. Endothelial Function and the Role of Ang II and Ang-(1-7) in Cardiac Remodeling

Cardiac remodeling can occur as a result of the increased tissue expression and activity of Ang II, which is triggered by harmful stimuli, such as hypertension and diabetes [39]. Ang II and Ang-(1-7) have opposing actions on cardiac myocytes [40]. Ang II can induce cardiac fibrosis by promoting growth in fibroblasts and myocytes, as well as activate the mitogen-activated protein (MAP) kinase cascade [22,41]. Furthermore, Ang II stimulates cardiac fibrosis by binding to AT1 receptors found in cardiac fibroblasts [41]. The interaction between Ang II and the AT1 receptor promotes cell proliferation [41] and collagen deposition through the upregulation of transforming growth factor beta (TGF-β) expression [42]. In contrast, Ang-(1-7) inhibits the growth of cardiac myocytes by exerting its effects through the functional Mas receptor [43]. In this regard, Tallant and colleagues [42] investigated the role of Ang-(1-7) in cultured neonatal rat myocytes. They found that the transfection of cultured myocytes with an antisense oligonucleotide targeting the Mas receptor blocked the Ang-(1-7)-mediated inhibition of serum-stimulated MAP kinase activation [39]. This finding supports the notion that Ang-(1-7) reduces the growth of cardiomyocytes by activating the Mas receptor. Another experimental study identified specific binding sites of Ang-(1-7) on adult rat cardiac fibroblasts [40]. The researchers discovered that this heptapeptide exhibits potential antifibrotic and antihypertrophic effects, which may counteract the actions of Ang II, suggesting a protective role in regulating cardiac remodeling [44]. 

Additionally, two experimental studies with human hearts have demonstrated that Ang-(1-7) is produced in the heart tissue. One study found that the production of Ang-(1-7) from both Ang I and Ang II was increased in failing human heart ventricles [45]. However, different enzymes were responsible for the conversion of Ang I and Ang II into Ang-(1-7), being, respectively, neutral endopeptidase and ACE2 [41]. The other study showed that Ang-(1-7) is also formed in intact human myocardial circulation and was found to be decreased when Ang II synthesis was suppressed [46]. These findings suggest that Ang-(1-7) can be mainly produced from Ang II to counterbalance its deleterious effects. The effects of Ang-(1-7) are beneficial and protective for heart functioning. 

**Figure 1:**
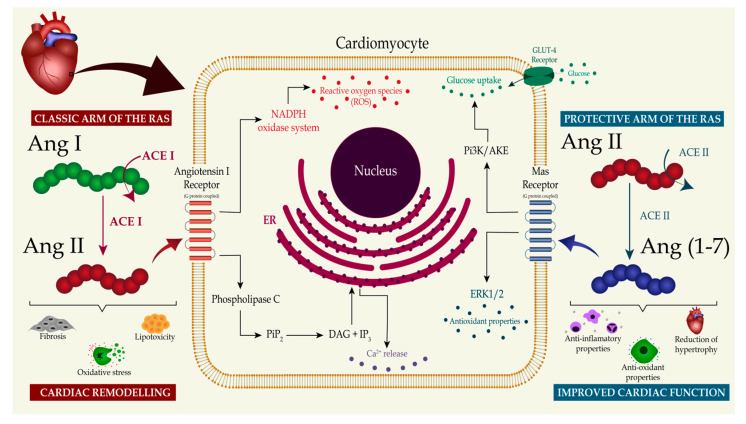
The RAS effects on the development of diabetic cardiomyopathy. This figure illustrates the effects of the two main regulatory arms of the renin–angiotensin system (RAS) on the development of diabetic cardiomyopathy. In the classic arm of the RAS, angiotensin-converting enzyme (ACE) converts angiotensin I (Ang I) into angiotensin II (Ang II), which activates angiotensin type 1 receptor (AT1). This pathway triggers calcium release and reactive oxygen species production, finally resulting in increased oxidative stress, lipotoxicity, and tissue fibrosis. Opposing these effects, in the protective arm of the RAS, angiotensin-converting enzyme 2 (ACE2) generates angiotensin-(1-7) (Ang-(1-7)) from Ang II. Ang-(1-7) initiates a downstream molecule cascade through Mas receptor activation that increases glucose uptake by cardiomyocytes and upregulates antioxidant molecules. The binding of Ang-(1-7) to the Mas receptor suppresses oxidative stress, inhibits tissue fibrosis, and prevents extensive cardiac remodeling. Abbreviations: NADPH—nicotinamide adenine dinucleotide phosphate hydrogen; PiP2—phosphatidylinositol 4,5-bisphosphate; DAG—diacylglycerol; IP3—inositol trisphosphate; Pi3K—phosphoinositide 3-kinase; ERK1—extracellular signal-regulated kinase 1.

The RAS is known to be tightly associated with the endothelial function and contributes to coronary-vessel vascular dynamics. The local production of Ang II, Ang-(1-7), adenosine, and nitric oxide, and the interplay between these molecules, regulate vasoconstriction and vasodilation in the heart [22]. Zhang and colleagues [46], in an experimental study on dogs with prediabetic metabolic syndrome, reported that the vasoconstriction produced by Ang II was abolished by the AT1 receptor blockade. The AT1 receptor blockade with telmisartan also improved the balance between coronary blood flow and myocardial oxygen consumption [43]. These findings suggest that coronary vasoconstriction mediated by AT1 receptor activation is augmented in prediabetic metabolic syndrome and contributes to the impaired control of the coronary blood flow through increased circulating Ang II and AT1 receptor expression. Conversely, four studies showed that Ang-(1-7) mediates coronary vasodilation [47,48,49,50]. The Ang-(1-7) vasodilatory effects can be attributed to the receptor-mediated mechanism and the bradykinin-induced release of nitric oxide [44,45,46,51]. Furthermore, an increase in circulating levels of Ang-(1-7) due to the prolonged administration of angiotensin-converting-enzyme inhibitors or Ang II receptor blockers may contribute to the cardioprotective actions of these medications [49]. In summary, Figure 1 illustrates the interplay between the two main regulatory arms of the RAS that contribute to causing or preventing cardiac remodeling and the important molecular pathways. 

Recent studies have highlighted the cardioprotective effects of the ACE2/Ang-(1-7)/Mas receptor axis. An experimental study aimed to investigate the effect of polyamidoamine (PAMAM) dendrimer nanoparticles on cardiac functioning following ischemia/reperfusion (I/R) injury in mammalian hearts. The study showed that PAMAM dendrimers can impair cardiac recovery after I/R injury in a dose-, dendrimer-generation-, and surface-charge-dependent manner. It was also found that the cardiotoxicity induced by PAMAM dendrimers can be mitigated by Ang-(1-7) acting through its MAS receptor. These findings suggest the activation of the Ang-(1-7)/MAS receptor axis as a potential strategy to overcome dendrimer-induced cardiotoxicity [52]. Another experimental study aimed to investigate the effects of an activator of ACE2, the compound diminazene aceturate (DIZE), in cardiomyopathy related to sepsis. The experimental model of sepsis was developed by cecal ligation and puncture. The treatment with DIZE mitigated the development of sepsis cardiomyopathy and preserved cardiac functioning during sepsis. The protective effects of DIZE were attributed to its interference with the renin–angiotensin system by promoting myocardial ACE2 expression and restoring local Ang-(1-7) levels [53]. The role of RAS components in sepsis-induced cardiomyopathy, specifically Ang II and Ang-(1-7), was also assessed in humans. The researchers measured the levels of Ang II and Ang-(1-7) in peripheral plasma samples obtained from healthy controls and septic patients and investigated the effects of exogenous Ang-(1-7) using in vitro and in vivo models. In summary, this study showed the dysregulation of the RAS with the predominance of Ang II and a reduction in Ang-(1-7) in patients with sepsis and supported the therapeutic potential of stimulating the ACE2/Ang-(1-7)/Mas receptor axis in attenuating myocardial damage associated with sepsis-induced cardiomyopathy [54]. Another study found beneficial effects of Ang-(1-7) in preventing adverse myocardial remodeling and extracellular matrix changes in the setting of cardiac overload. The experimental models included normotensive and hypertensive rats, and Ang-(1-7) transgenic rats that underwent aortocaval fistulas to induce volume overload. Biometric and heart tissue analyses were performed after five weeks. Based on these findings, the study concluded that Ang-(1-7) exhibited cardioprotective and antifibrotic potential in the context of cardiac volume overload. The peptide attenuated cardiac hypertrophy, reduced fibrosis, and modulated matrix-remodeling pathways. Furthermore, Ang-(1-7) increased the expression of Cx43, suggesting a potential positive impact on electrical coupling between cardiomyocytes [55].

## 4. Renin–Angiotensin System and Its Associations with Type 2 Diabetes Mellitus

The pathogenesis of T2DM is mainly centered on the impaired glucose uptake by cells, a feature known as insulin resistance. The dysregulation and overactivity of the RAS play critical roles in the development of insulin resistance. The demonstration of the production of RAS components and their activity in local environments in an organism by Ganten and colleagues [50] has led to an improved subsequent understanding of its actions on metabolic pathways in different organs and systems [56,57,58]. The RAS has autocrine, paracrine, and endocrine functions [59]. There is a complex interaction between systemic and local RASs with cells and tissues that contributes to vascular and endothelial homeostasis [35,60]. Multiple mechanisms have also resulted in insulin resistance [61].

### 4.1. Insulin Resistance

Among the RAS components implicated in the pathophysiology of insulin resistance, Ang II activity plays a prominent role in the dysregulation of glucose homeostasis [61]. As an RAS molecule that is present in skeletal-muscle myocytes, Ang II actions on skeletal muscle contribute to local insulin resistance mainly through two independent pathways: insulin signaling/insulin-mediated GLUT-4 glucose uptake and alterations in blood flow [61,62,63]. It should be mentioned that the Ang II effects on insulin signaling and glucose uptake are completely independent of any vasoconstrictive actions of the peptide [64]. Two studies with rats whose skeletal-muscle myocytes were exposed to an in vivo infusion of Ang II and the incubation of the peptide in cell culture found effects on insulin resistance [63,65]. The results showed decreased GLUT-4 translocation, impaired glucose uptake, increased oxidative stress [65], and inhibited insulin signaling via the decrease in the phosphorylation of glycogen synthase kinase 3 beta Ser (9) and AKT Ser473 [63]. Regarding the hemodynamic effects of Ang II, an experimental study [64] measuring the microvascular blood volume in rats during the systemic infusion of the peptide revealed that angiotensin 1 receptor (AT1R) activation restricts the microvascular blood volume and glucose extraction. Early studies conducted on human subjects [66] and dogs [67] demonstrated that the systemic infusion of Ang II produced variations in glucose utilization due to the negative relationship between the peptide and microvascular blood flow, further contributing to insulin resistance. 

A third mechanism of Ang II-mediated insulin resistance should also be considered. At a cellular level, Ang II induces oxidative stress via the activation of AT1R and NADPH oxidase, resulting in reactive oxygen species generation and consequently contributing to insulin resistance [68,69]. Blendea et al. [68], in a study comparing the effects of the Ang II receptor blocker (ARB) valsartan and a superoxide dismutase mimetic on whole-body glucose tolerance and skeletal-muscle insulin-stimulated glucose uptake in rats, demonstrated improvements in the skeletal-muscle insulin-dependent glucose uptake and whole-body insulin resistance. An in vitro study with mammalian skeletal muscle showed that oxidative stress leads to the substantial insulin resistance of distal insulin signaling and glucose transport activity [70]. These changes were associated with a selective loss of the insulin receptor substrate-1 (IRS-1) protein and a p38 MAPK-dependent mechanism [70]. Together, these findings support the negative effect of oxidative stress on glucose metabolism and insulin signaling. 

The local pancreatic RAS directly modulates insulin secretion via beta cells in the pancreas [71]. The effect of Ang II on insulin secretion has been shown in animal and human studies. In rats [71], the blood flow to the pancreas was determined after local Ang II infusion and showed a reduction in the islet blood flow and delayed insulin release in response to glucose. Another study [72] analyzing the effect of RAS inhibitors found that captopril and pravastatin enhanced insulin secretion and positively influenced glycemia in intraperitoneal glucose tolerance tests. These findings suggest that the local pancreatic RAS significantly influences blood flow and contributes to insulin homeostasis. In a randomized, double-blind, controlled clinical trial, the effects of 26 weeks of valsartan on beta-cell functioning and insulin sensitivity were assessed [73]. Compared to the placebo, the valsartan treatment increased the glucose-stimulated insulin release and sensitivity in individuals with impaired glucose metabolism. A multicenter trial conducted on obese adults aimed to compare the effects on glucose metabolism of combined hydrochlorothiazide and valsartan therapy versus hydrochlorothiazide and calcium-channel-blocker (amlodipine) treatment [74]. The study concluded that the combination of valsartan and hydrochlorothiazide was associated with greater glucose-stimulated insulin secretion [74]. Considering these findings, we can conclude that the local pancreatic RAS can negatively regulate insulin availability through local vasoconstriction mechanisms, contributing to diabetes pathogenesis. Conversely, ACE inhibitors and ARBs have beneficial effects on the pancreas, enhancing insulin release. 

Obesity has been demonstrated to have an extensive relationship with insulin resistance, and RAS activity within adipose tissue is linked to its pathogenesis. It is known that, as the adipose tissue mass increases, the local expression of the RAS components is enhanced alongside [75]. Ang II plays an essential role in preventing fat-cell differentiation, thereby contributing to the formation of larger, already existing adipocytes [76]. These dysfunctional adipocytes increase lipolytic activity, pro-inflammatory adipokines, cytokine secretion, and oxidative stress. Globally, the dysfunction enhances lipolysis and contributes to insulin resistance [76]. Two experimental studies extracted fat tissue cells from both rats [77] and humans [78] and exposed the adipocytes to Ang II. It was found that Ang II contributes to an increased adipocyte size. In humans, the blockade of the RAS has been demonstrated to be beneficial in individuals with impaired glucose metabolism. In a randomized, double-blind, placebo-controlled study, valsartan treatment markedly reduced the abdominal subcutaneous adipocyte size and macrophage infiltration markers and increased the blood flow to the adipose tissue overall, suggesting that interventions targeting the RAS may improve the adipose tissue function [79]. A smaller study assessing glucose homeostasis and adipose tissue inflammatory markers following a period of valsartan treatment showed similar findings, with improved insulin sensitivity and associated low-grade inflammation after ARB treatment [73]. 

Apart from Ang II, aldosterone and Ang-(1-7) also play essential roles in modulating insulin resistance. Aldosterone excess has been linked to diabetes since the 1960s [80,81]. Aldosterone acts in a mineralocorticoid-receptor-dependent manner to impair insulin sensitivity in adipocytes and skeletal muscle. The hormone impairs glucose-induced insulin secretion in pancreatic islets due to reactive oxygen species production in a receptor-independent manner [82] and interferes with the insulin signaling pathway by downregulating IRS-1 in vascular-smooth-muscle cells [83]. Many clinical studies have suggested that aldosterone is associated with impaired insulin sensitivity in humans. In four cross-sectional observational studies [84,85,86,87], insulin sensitivity was assessed by indirect parameters. Insulin sensitivity was diminished in patients with primary hyperaldosteronism, supporting a more direct association with aldosterone, as Ang II is low in this disease. In two of these studies, patients were treated with either adrenalectomy [86,87] or a mineralocorticoid receptor antagonist (spironolactone) [86]. In both cases, the reduction in the aldosterone levels induced the regression of glucometabolic complications. A study comparing the effects of chlorthalidone plus spironolactone versus chlorthalidone plus irbesartan (an angiotensin receptor 1 (ATR1) inhibitor) supports the isolated effect of aldosterone on insulin resistance. Chlorthalidone alone or in combination with irbesartan increased the indices of insulin resistance. However, this finding did not occur when chlorthalidone was administered in combination with spironolactone [88]. In contrast to these results, two cross-sectional observational studies [89,90] comparing primary hyperaldosteronism patients to controls with essential hypertension found no significant differences in the frequency of diabetes mellitus, insulin resistance, or impaired glucose tolerance between the groups. Overall, the blockade of the mineralocorticoid receptor appears beneficial in improving insulin resistance when aldosterone levels are elevated, highlighting the role of aldosterone and mineralocorticoid receptor activation in contributing to insulin resistance. 

### 4.2. Endothelial Function and Oxidative Stress

In contrast to Ang II and aldosterone, Ang-(1-7) exerts beneficial effects in insulin homeostasis. Ang-(1-7) is a byproduct of Ang II produced by angiotensin-converting enzyme 2 (ACE2) and acts via the G-coupled receptor Mas [91]. Ang-(1-7) antagonizes the vasoconstrictive actions of Ang II, promoting the release of NO and prostaglandins, being preferentially a vasodilator peptide [48,91]. Ang-(1-7) is significantly involved in the maintenance of endothelium integrity. Both animal and human studies have demonstrated that Ang-(1-7) improves insulin sensitivity and glucose metabolism [63]. However, the number of studies in humans assessing the role of Ang-(1-7) in glucose homeostasis is still limited. An observational study comparing pregnant women with and without gestational diabetes [92] that measured serum levels of Ang I, Ang II, and Ang-(1-7) showed reduced circulating levels of Ang-(1-7) in individuals with gestational diabetes. This result suggests that reduced levels of the vasodilatory molecule could be implicated in the endothelial dysfunction seen in gestational diabetic women during and after pregnancy and a possible factor implicated in the pathogenesis of insulin resistance during pregnancy. Additionally, a larger study [93], including 859 patients with type 1 diabetes mellitus, assessed the ACE2 activity in serum samples. The results showed that the ACE2 activity was increased in men with diabetes and microalbuminuria compared to those without albuminuria, indicating that the high activity of ACE2 may be a compensatory mechanism to regulate the vascular and renal functions in patients with diabetes. In experimental studies, ACE2 [94] or Mas receptor [95] knockout mice showed decreased insulin sensitivity compared to wild-type animals. Ang II infusion or a high-fat, high-sucrose diet impaired glucose tolerance and insulin sensitivity more severely in knockout mice than wild-type animals. In addition, the infusion of Ang-(1-7) enhanced glucose tolerance [94]. More studies in this field are needed, but data suggest an essential role of the ACE2/Ang-(1-7)/Mas axis in improving insulin resistance and antagonizing the actions of the ACE/Ang II/AT1R axis. 

Overall, enough evidence supports the association between the overactivity of the ACE/Ang II/AT1R axis and T2DM onset. In this regard, essential findings in large-scale clinical trials suggest that RAS inhibition benefits glucose homeostasis. At the beginning of the century, there was no consensus on the recommendation of the most suitable antihypertensive drug to reduce the risk of cardiovascular disease in patients with hypertension and diabetes. Therefore, the LIFE (Losartan Intervention For Endpoint Reduction in Hypertension) study was conducted to compare the effects of losartan and atenolol [96]. The LIFE study included 1195 patients with diabetes, hypertension, and left-ventricular hypertrophy randomized into two groups receiving an ARB or beta blocker. The results showed that the use of losartan was associated with a reduction in the occurrence of new diabetes onset when compared with atenolol.

Similarly, a more extensive study named HOPE (Heart Outcomes Prevention Evaluation) [97], including 9297 high-risk patients who had evidence of vascular disease or diabetes, also demonstrated that the diabetes incidence was lower in the group treated with an ACE inhibitor, ramipril, when compared with the placebo. More recent studies have shown similar findings. The NAVIGATOR (Nateglinide and Valsartan in Impaired Glucose Tolerance Outcome Research) study [98] assigned 9306 patients with impaired glucose tolerance and cardiovascular disease or risk factors to receive valsartan or a placebo and analyzed the diabetes development rate for a median of 5 years. The study showed a lower incidence of diabetes in the ARB-treated group compared with the placebo group. Additionally, the DREAM (Diabetes Reduction Assessment with Ramipril and Rosiglitazone Medication) study [99] randomly assigned 5269 participants with impaired fasting glucose levels or impaired glucose tolerance to receive ramipril or placebo. The subjects were followed for a median of 3 years. The authors reported significantly lower plasma glucose levels in response to the glucose load in the group treated with ramipril but no reduction in the incidence of diabetes. 

Together, these findings support the benefits of the RAS blockade on glucose homeostasis using ACE inhibitors and ARBs. RAS inhibition has been shown to improve insulin sensitivity and reduce the incidence of new T2DM in high-risk individuals [96,97,98,99].

## 5. Diabetic Cardiomyopathy

In patients with the diagnosis of T2DM, the natural history of the disease is marked by the affliction of the kidneys and the heart, which is mediated and modulated by alterations in the RAS. Among the chronic complications of persistent elevated glycemic values, diabetic cardiomyopathy is one important, but overlooked, manifestation [100,101]. The pathogenesis of this complication begins with the negative effects of the excessive amount of glucose in the tissues, as the advanced glycation end products are extremely pro-inflammatory and, therefore, harmful to the cardiomyocytes and endothelium. Additionally, because of the alterations in the lipidic metabolism, a fatty acid accumulation in the intracellular compartment occurs and, hence, this glucotoxicity culminates in the formation of reactive oxygen species, which are strongly damaging to the cells [102,103].

The combination of these metabolic alterations results in the death of the cardiomyocytes, the fibrosis of the cardiac tissue, and an overall inflammatory state of the organism. In consequence, the heart suffers hypertrophy and a decrease in its complacency, leading to systolic and diastolic dysfunction [102,104,105]. This entire process is enhanced by the exacerbated activation of the classical RAS axis in situations of metabolic disarrangements. The levels of angiotensinogen are increased due to peripheral production by the adipocytes. Consequently, the synthesis of Ang II is enhanced, which, in turn, stimulates inflammation and oxidative stress [106]. High concentrations of Ang II also contribute to the progression of diabetes itself, as this peptide is associated with augmented insulin resistance and damage to the pancreatic tissue [69,107].

Furthermore, the renal function impairment due to glomerular sclerosis results in chronic kidney disease, culminating in increases in the neurohormonal modifications previously cited [108]. Consequently, the inflammation responsible for the development of heart failure in this group of patients intensifies. The pathophysiological association between heart and kidney damage is called cardiorenal syndrome type 4 and is a predictor of mortality in patients with chronic diseases that afflict the kidneys. Therefore, a vicious cycle occurs, as the activation of the classical RAS axis leads to metabolic alterations involved in T2DM, which chronically affect the kidney and heart tissues [108,109].

The ACE-2/Ang-(1-7)/Mas axis has an important counterregulatory effect as a depressor arm of the RAS, exerting cardioprotective and nephroprotective actions [14]. In vitro studies have demonstrated beneficial effects of this axis. One important study [110] assessing the effects of Ang-(1-7) treatment on diabetic cardiomyopathy in mice demonstrated that Ang-(1-7) infusion improved cardiac hypertrophy and diastolic dysfunction, also suppressing myocardial oxidative stress. Knockout mice with the deletion of the ACE2 gene exhibited reduced systolic function [111]. The infusion of recombinant human ACE2 in these mice attenuated the pressure-overload-induced adverse myocardial remodeling and angiotensin II effects on heart tissue [86]. These findings highlight ACE2 as an important protector molecule in diabetic-induced heart disease, suppressing adverse myocardial remodeling. Therefore, the upregulation of the ACE-2/Ang-(1-7)/Mas axis may represent a potential target for diabetic cardiomyopathy and heart failure treatment. However, additional clinical studies with human subjects are required. 

Beyond its cardioprotective effects, the ACE-2/Ang-(1-7)/Mas axis has also been demonstrated to display nephroprotective properties in diabetes models. In a study performed in murine models, treatment with Ang-(1-7) significantly reduced the glomerular area, mesangial expansion, and oxidative stress [112], showing beneficial effects of Ang-(1-7) treatment on the renal function. One study [113] conducted with diabetic patients with chronic kidney disease found that the treatment with olmesartan significantly increased the urinary ACE2 levels independently of the blood pressure and plasma aldosterone levels and reduced the albuminuria and plasma aldosterone levels. Olmesartan may have the unique effect of increasing urinary ACE2 levels, which may contribute to a preserved renal function. Table 1 summarizes the important studies and their main findings discussed in this section.

Despite the evidence of the beneficial effects of the ACE2/Ang-(1-7)/Mas axis in heart function and experimental models of diabetic cardiomyopathy, very few studies included humans. Most studies with patients only measured the molecules in plasma or urine. Whether the same beneficial effects of the stimulation of the ACE2/Ang-(1-7)/Mas axis observed in experimental studies occur in patients with diabetic cardiomyopathy still remains to be evaluated. Thus, the usefulness of ACE2 activation and Ang-(1-7) administration in clinical practice needs to be investigated in the future. 

## 6. Conclusions

In summary, the RAS plays an important role in the development and progression of diabetic cardiomyopathy. The over-activation of the classical RAS axis in diabetes leads to the increased production of Ang II, AT1 receptor activation, and aldosterone release, contributing to increased oxidative stress, fibrosis, and cardiac remodeling. The ACE-2/Ang-(1-7)/Mas receptor axis counterbalances the deleterious effects of the ACE/Ang II/AT1 receptor axis by means of cardioprotective and nephroprotective actions. The binding of Ang-(1-7) to the Mas receptor suppresses oxidative stress, inhibits tissue fibrosis, and prevents extensive cardiac remodeling. Several studies have demonstrated that the inhibition of the classical RAS axis can improve outcomes in diabetic cardiomyopathy. The use of ACE inhibitors and ARBs improves heart function and reduces the risk of cardiomyopathy development in patients with diabetes. Experimental studies also support a beneficial effect of Ang-(1-7) and human recombinant ACE2 infusion in diabetic cardiomyopathy. Despite the potential benefits of these endogenous molecules, further research is necessary to translate experimental findings into clinical practice.

The ACE2/Ang-(1-7)/Mas receptor axis counteracts the classical ACE/Ang II/AT1 receptor axis and inhibits other signaling pathways involved in inflammation, fibrosis, and oxidative stress. Further studies are needed to understand the complex crosstalk between these pathways and determine the net effect on cardiovascular functioning and pathology. The therapeutic potential of modulating the ACE2/Ang-(1-7)/Mas receptor axis is an area of active research. However, there is a need for more studies to explore the efficacy and safety of targeting this axis for the treatment of cardiovascular diseases, such as hypertension, heart failure, and vascular disorders. Understanding the clinical implications of the ACE2/Ang-(1-7)/Mas receptor axis is crucial. This includes assessing the diagnostic and prognostic value of the ACE2 levels, identifying patient populations that may benefit from therapies targeting this axis, and determining the optimal strategies for modulating ACE2/Ang-(1-7)/Mas receptor activity in clinical practice. Overall, addressing these knowledge gaps will enhance our understanding of the ACE2/Ang-(1-7)/Mas receptor axis and its therapeutic potential in cardiovascular diseases, potentially leading to the development of novel treatment strategies.

## Figures and Tables

**Table 1:** Main findings of studies on diabetic cardiomyopathy.

Author	Reference Number	Study Design	Sample	Main Findings	Conclusion/Knowledge Gaps
Mori et al.	[110]	Experimental	Mice	Angiotensin-(1-7) reduced cardiac hypertrophy, lipotoxicity, and adipose tissue inflammation. The heptapeptide also produced the upregulation of adipose triglyceride lipase and completely rescued the diastolic dysfunction.	Ang-(1-7) represents a promising therapy for diabetic cardiomyopathy associated with type 2 diabetes mellitus.
Crackower et al.	[111]	Experimental	Mice	Targeted disruption of angiotensin-converting enzyme 2 (ACE2) resulted in severe cardiac contractility defect, increased angiotensin II levels, and produced the upregulation of hypoxia-induced genes in the heart. Genetic deletion of angiotensin-converting enzyme (ACE) on an ACE2 mutant background completely recovered the cardiac dysfunction.	Genetic data for ACE2 show that the enzyme is an essential regulator of heart function in vivo.
Papinska et al.	[112]	Experimental	Mice	An improvement in heart function was observed in Ang-(1-7)-treated mice. Reduced cardiomyocyte hypertrophy, fibrosis, and inflammatory-cell infiltration were also observed. These changes were blocked by antagonists of the MAS1, AT2, and bradykinin receptors.	Short-term administration of angiotensin-(1-7) in young db/db mice improved heart function and reduced kidney damage.
Abe et al.	[113]	Open-label, interventional study	31 individuals	The treatment with olmesartan increased urinary ACE2 levels and reduced albuminuria, urinary liver-type fatty acid binding protein (L-FABP), and plasma aldosterone levels.	Olmesartan may increase urinary ACE2 levels. However, whether this contributes to olmesartan’s renoprotective effect must be examined further.

## Data Availability

Not applicable.
